# Pharmacological Characteristics of Porcine Orexin 2 Receptor and Mutants

**DOI:** 10.3389/fendo.2020.00132

**Published:** 2020-03-31

**Authors:** Min Liu, Tianqi Min, Haijie Zhang, Yuan Liu, Zhiqiang Wang

**Affiliations:** ^1^College of Veterinary Medicine, Yangzhou University, Yangzhou, China; ^2^Jiangsu Co-innovation Center for Prevention and Control of Important Animal Infectious Diseases and Zoonoses, Yangzhou, China; ^3^Institute of Comparative Medicine, Yangzhou University, Yangzhou, China

**Keywords:** orexin 2 receptor, pig, mutations, cAMP, signaling pathway

## Abstract

Orexin receptors (OXRs) play a critical regulatory role in central control of food intake, maintenance of sleeping states, energy metabolism, and neuroendocrine homeostasis. However, most previous studies have focused on the sleep-promoting functions of OXRs in human beings, while their potential value in enhancing food intake for livestock breeding has not been fully exploited. In this study, we successfully cloned porcine orexin 2 receptor (pOX2R) complementary DNA and constructed four pOX2R mutants (P10S, P11T, V308I, and T401I) by site-directed mutagenesis, and their functional expressions were further confirmed through Western blotting analysis. Pharmacological characteristics of pOX2R and their mutants were further investigated. These results showed that the P10S, P11T, and T401I mutants had decreased cAMP signaling with orexin A, whereas only the P11T mutant decreased under the stimulation of orexin B. Besides, only P10S displayed a decreased calcium release in response to both orexin ligands. Importantly, these mutants exhibited decreased phosphorylation levels of ERK1/2, p38, and CREB to some degree compared with wild-type pOX2R. Collectively, these findings highlight the critical role of these mutations in pOX2R signaling and expand our understanding of molecular and pharmacological characterization of pOX2R.

## Introduction

Orexins involve orexin A and B are a pair of lateral hypothalamic neuropeptides that were originally identified as the endogenous ligands for two G-protein coupled receptors, namely orexin receptors 1 (OX1R) and 2 (OX2R) (1). Orexin A (OXA), a *C*-terminally amidated 33-residue peptide with two intramolecular disulfide bridges and an *N*-terminal pyroglutamate residue, shows similarly high potency for both OX1R and OX2R (2). Of note, the peptide sequences of orexin A are usually highly conserved in human and other animals. Orexin B (OXB), a 28-amino-acid and *C*-terminally amidated linear hydrophilic peptide, exhibits higher binding activity to OX2R than OX1R, with about 10-fold difference. Unlike OXA, there are differences in the OXB derived from different animals, such as the substitution of serine at position 2 and/or 18 in human by proline and/or glutamine in other mammals (3). OX1R and OX2R belong to the rhodopsin-like receptor family and show 64% identical amino acid sequences. Recent crystal structure analysis demonstrated that human orexin receptor 2 (hOX2R) lacks a conserved amphipathic α-helix in the extracellular *N*-terminal region compared to hOX1R (4, 5). It has also been proved that the interaction of Orexins and OX1R/OX2R would activate the three subunits of the Gα protein, which in turn regulates various phospholipases, non-selective cation channels, adenylate cyclase, proteins, and lipid kinases, and finally modulates downstream signaling (6–8). Specifically, the stimulation of OXA on OX1R was found to trigger the release of 2-arachidonic acid, which further activates p38 and ERK1/2 in a time- and concentration-dependent manner, and the increased phosphorylation is closely related to Gq/PLC/PKC signaling but is not involved in the PKA pathway (9–11).

The physiological functions of orexin receptors, including central control of food intake, regulation of sleep and wakefulness (12, 13), energy metabolism (14, 15), and neuroendocrine homeostasis (16), have already been fully demonstrated. For example, mice that showed high levels of orexins could obtain more weight increase under the same feeding conditions (17). In contrast, orexin-deficient mice exhibited loss of appetite (18). Under restricted feeding conditions, the expression of orexins in animals significantly increased and further facilitated the intake of food (19). In addition to food intake, orexins and receptors were also found to participate in the regulation of sleep (20). For instance, people with narcolepsy often have lower levels of orexins in cerebrospinal fluid (21). This suggests that the inhibition of orexin receptors in human may contribute to the alleviation of insomnia (22). Indeed, an effective small-molecule antagonist of orexin receptors termed Suvorexant (23) was approved by the FDA for the treatment of insomnia in 2014 (24). Despite these ongoing efforts in human, the pharmacological characteristics and functions of orexin receptors in pig are still largely unknown. Considering that the OX1 receptor is mainly involved in motivation/reward (25) and the OX2 receptor is closely related to the modulation of the sleep/wake cycle, energy homeostasis (26, 27), and food intake (28), we reasoned that the OX2R in pig may be closely associated with the rate of weight gain. As is well-known, the pig is the most critical economic animal in China (29), and the rate of weight gain is an important indicator of economic traits. Therefore, promoting their breeding and increasing animal weight are feasible measures to accelerate the development of the aquaculture industry. However, the pharmacology and functional characterization of pOX2R and its mutations have still not been fully studied.

In this study, we first successfully cloned pOX2R and constructed four mutations thereof, namely P10S, P11T, V308I, and T401I. Besides, we investigated the intracellular cAMP generation of these receptors under the stimulation of OXA and OXB through dual-luciferase reporter gene assay. Meanwhile, the phosphorylation levels of extracellular regulated protein kinases 1/2 (ERK1/2), p38, and cAMP-response element-binding protein (CREB) induced with two agonists were determined through Western blotting analysis. These results showed that OXA and OXB would lead to increased intracellular cAMP and calcium release in a dose-dependent manner for both wild type and four mutants. However, the P11T mutation significantly decreased cAMP production, while the P10S mutation remarkably reduced the calcium release with two ligands. In addition, we observed significantly increased phosphorylation levels of ERK1/2, p38, and CREB in wild-type pOX2R with OXA/OXB, whereas most mutations exhibited decreased phosphorylation levels. Taken together, these results demonstrated that these four mutations in pOX2R have a potential effect on downstream protein phosphorylation and related physiological functions.

## Materials and Methods

### Materials

Expression vector pcDNA3.1(+) was purchased from Invitrogen (Carlsbad, CA, USA). A fast mutagenesis system kit was purchased from Transgen Biotech (Beijing, China). An Exfect 2000 transfection reagent was obtained from Vazyme Biotech (Nanjing, China). DMEM/F-12 1:1 medium was purchased from Thermo Fisher Scientific (Beverly, MA, USA). Reporter gene plasmids pGL4.29[luc2P/CRE/Hygro] and pGMLR-TK were purchased from Promega (Beijing, China). A dual-luciferase reporter gene assay kit was purchased from Beyotime Biotechnology (Shanghai, China). PMSF and protease inhibitors were purchased from Solarbio Life Science (Beijing, China). c-myc Rabbit mAb was obtained from Abcam (Cambridge, UK). Goat anti-rabbit IgG-HRP was purchased from Cell Signaling Technology (Boston, MA, USA).

### Molecular Cloning of pOX2R

The pOX2R coding DNA sequence was amplified directly from pig genomic DNA using sense primer 5′- CCCAAGCTTATGTCCGGCACCAAACTGGAGGAC-3′ and antisense primer 5′- GCTCTAGACTACCAGTTTTGGAGCTGCCCCGC-3′ based on the published nucleotide sequence in the NCBI database (NM 001129951.1), incorporating HindIII and XbaI restriction sites in sense and antisense primers (underlined), respectively. PCR amplification was performed based on the following cycling parameters: 5 min at 94°C for one cycle, followed by 30 s at 94°C, 30 s at 60°C, and 60 s at 72°C for 30 cycles, then a final extension at 72°C for 5 min. After amplification, PCR products were separated and visualized by agarose gel electrophoresis with ethidium bromide. Correct PCR products were double-digested with HindIII and XbaI (Promega, Shanghai, China) and ligated into the expression vector pcDNA3.1(+) using T4 DNA ligase (Trans-Gen Biotech) at 16°C overnight.

Recombinant plasmids were transformed into competent *Escherichia coli* DH5α cells, and cells were grown overnight on an LB-agar plate containing 50 μg/ml ampicillin. Plasmid DNA of single colonies was extracted using a mini-preparation kit (Axygen Biosciences, CA, USA) after digestion with HindIII and XbaI. The nucleotide sequence of the cloned *pOX2R* was determined by DNA sequencing, and Myc tag (AAGCTGATCTCAGAAGAAGACCTATCCGGC) was added at the *N*-terminus (Sangon Biotech, Shanghai, China). Plasmid DNA containing a Myc epitope tag and the pOX2R sequence (myc-pcDNA3.1-pOX2R) was extracted using a Plasmid Maxi kit (Axygen Biosciences, CA, USA).

### Phylogenetic Analysis and Homology Models

The phylogenetic relationship of cloned *pOX2R* and other related genes from the NCBI database, including from human, mouse, rat, cattle, sheep, dog, cat, chicken, and zebrafish (GenBank accession number or NCBI reference sequence number: NM_001526.4, NM_001364551.1, NM_013074.1, NM_001192677.1, XM_004018732.4, NM_001002933.1, XM_019830741.1, NM_001024584.1, and NM_001079868.1, respectively) were compared. Multiple alignment of selected sequences was conducted by ClustalX 2.1. Then, a maximum likelihood tree was produced with MEGA 6.0 (30). The reliability of the resulting trees was evaluated by bootstrapping with 1000 replications. Lastly, phylogenetic trees were visualized with iTOL (31). For homology models of OX2Rs, SWISS-MODEL was used to perform protein 3D structure prediction. The structural figures were embellished by PyMOL (32).

### Site-Directed Mutagenesis

Cloned wild-type pOX2R tagged with c-Myc at the *N*-terminus based on a previous report (33) was used as the template for subsequent mutagenesis. Four-point mutations were constructed through designed primers (Table S1) with a site-directed mutagenesis system kit (Transgen Biotech, Beijing, China). In addition, the nucleotide sequences of four pOX2R variants purified from the single clone were determined by DNA sequencing to confirm the presence of the correct mutations. Plasmid DNA containing four pOX2R mutations was prepared by Plasmid Maxi kit (Axygen Biosciences, CA, USA) for subsequent transfection.

### Cell Culture and Transfection

Human embryonic kidney (HEK) 293T cells, purchased from the American Type Culture Collection (Manassas, VA, USA), were grown in a culture medium, containing 10% newborn calf serum, 10 units/mL penicillin, and 0.1 mg/mL streptomycin at 37°C in an incubator in 5% CO_2_. For cAMP assays, 5.0 × 10^5^ HEK293T cells were transiently transfected with pOX2Rs and the reporter gene plasmids, including firefly and renilla luciferase reporter plasmid pGL4.29[luc2P/CRE/Hygro] and pRL-TK reporter plasmid (pOX2R: pGL4.29: pGMLR-TK of 2:10:1), using ExFect 2000 reagent according to the manufacturer's instructions. For Western blotting analysis, 5.0 × 10^5^ cells were transiently transfected with pOX2R wild type or mutant plasmids alone.

### cAMP Assays

cAMP levels were determined using a dual-luciferase reporter gene assay kit (Beyotime Biotech, Shanghai, China) according to the manufacturer's instructions. Briefly, after transient transfection for 24 h (described in section Cell Culture and Transfection), transfected HEK293T cells expressing pOX2R or the mutants were cultured into 24-well plates and stimulated with buffer containing either 10^−6^–10^−10^ mol/L orexin A or orexin B at 37°C for 9 h. Then, cells were washed twice with DPBS and lysed with 200 μL lysis buffer. A 20-μL volume of cell lysate was mixed with 100 μL Luciferase Assay Reagent. Determination of the illumination for each treatment was immediately performed on a GloMax-Multi microplate reader (Promega, Madison, WI, USA). All tests were performed in triplicate, EC_50_ (concentration of ligand that causes 50% maximal cAMP production) values and Rmax (maximal response) were calculated using Graphpad Prism 6.0 software.

### Intracellular Calcium Determination

Intracellular calcium levels were detected using the Fluo-4 AM assay kit (Beyotime Biotech, Shanghai, China) according to the manufacturer's instructions. Briefly, transfected HEK293T cells at a density of 10^5^ cells/well were cultured for 24 h and stimulated with orexin A or orexin B. Subsequently, cells were washed twice with assay buffer, and 100 μL loading dye solution was added. Cells were incubated at 37°C for 30 min and then at room temperature for an additional 30 min. Fluorescence was measured using an Infinite M200 Microplate reader (Tecan, Mannedorf, Switzerland) with an excitation wavelength of 485 nm and an emission wavelength of 525 nm.

### Western Blotting Analysis

Phosphorylation levels of ERK1/2, p38, and CREB were detected by Western blotting. Transfected HEK293T cells processed as detailed in section Site-Directed Mutagenesis were cultured in a six-well plate and were grown to 70–80% confluency. After 24 h of serum starvation, the cells were treated with either 10^−7^ M orexin A or 10^−6^ M orexin B for 5 min (p-ERK1/2) or 30 min (p-p38 and p-CREB) based on our preliminary experiments. Following treatment, cells were washed with ice-cold PBS and immediately lysed in lysis buffer containing a 1:50 dilution of protease inhibitor cocktail and a 1:100 dilution of PMSF (Solarbio Life Science, Beijing, China). The cell lysates were centrifuged at 10,000 rpm at 4°C for 10 min. The supernatant was transferred into a new tube and then stored at −20°C. Proteins were separated by 12% SDS-PAGE acrylamide gel electrophoresis and transferred onto PVDF membranes at 200 V for 30 min. Non-specific binding was reduced by incubating membranes for 2 h in blocking buffer [5% non-fat milk in TBS/Tween 20 (0.1%)] at RT. Primary antibodies (Cell Signaling Technology, Danvers, MA, USA) were diluted with 5% non-fat milk in TBS/Tween 20 (0.1%) (1:3,000) and incubated with the membrane at 4°C overnight with shaking. The membrane was washed three times with 0.1% Triton X-PBS for 10 min, and the secondary antibody (Cell Signaling Technology, Danvers, MA, USA) was diluted 1:10,000 in TBST (Tris buffer saline with 0.1% Tween-20) and incubated with membrane with shaking. Finally, protein bands were detected using NcmECL Ultra Reagent (New Cell & Molecular Biotech, Suzhou, China) and quantified by ImageJ software.

### Data Analysis

Digital images of Western blots were analyzed by densitometry using Image J, and the dose-response analysis was performed using GraphPad Prism 6 software. Each result represents the mean ± SEM of at least three experiments. Unpaired *t*-test between two groups or one-way ANOVA among multiple groups were used to calculate *P*-values (^*^P < 0.05, ^**^P < 0.01, ^***^P < 0.001).

## Results

### Phylogenetic and Protein Structure Analysis

**Figure 1:**
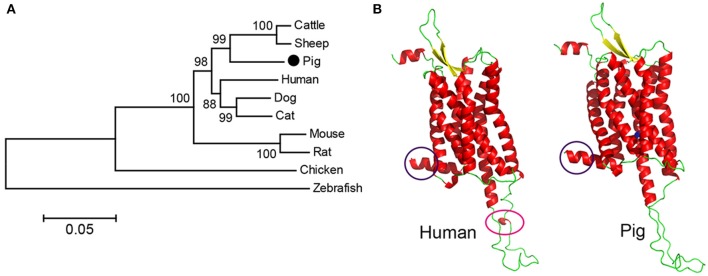
Phylogenetic analysis and homology model of cloned *pOX2R*. **(A)** Phylogenetic tree of OX2R nucleotide sequences from 10 species. **(B)** Comparison of predicted protein structures of OX2Rs from human and pig. The significant differences are marked by purple or pink circles. Valine at position of 308 in pOX2R is labeled in green.

According to the putative *pOX2R* gene in the NCBI database, we designed primers to amplify the full-length *pOX2R* sequence (described in section Molecular Cloning of pOX2R). To compare the similarity of *OX2R* genes from different species, nucleotide sequence alignment of *OX2R* genes from human, mouse, rat, cattle, sheep, dog, cat, chicken, and zebrafish was performed. As shown in Figure 1A, we found that *OX2R* from pig showed higher homology with cattle and sheep (99% similarity) compared with other species. Unsurprisingly, the lowest similarity with zebrafish was observed due to kinship being distant. To better evaluate the differences between hOX2R and pOX2R in whole conformation level, homology modeling was performed based on their amino acid sequences (Figure 1B). Although OX2R from human and pig showed high spatial structural similarity, there are still some differences between them. For example, intracellular parts (lower structure) in hOX2R possess an additional α-helix compared with pOX2R.

### Construction of Mutants and Basal Activity Determination in HEK293T Cells

To investigate the role of some key amino acids in pOX2R cellular signaling, we constructed four mutants that have been found to be critical in human beings (34) (see Discussion section), namely P10S, P11T, V308I, and T401I (Figure S1). To further evaluate the expression of pOX2R and its four mutants in HEK293T cells, the Myc sequence at the *N* terminal as a label and eukaryotic expression vector (pcDNA3.1) were uploaded, and corresponding Western blotting analysis was performed. The results showed that both wild type and the four mutants could successfully express about 50 kDa protein, whereas no expressed protein was detected in empty vector and non-transfected cells (Figure S2). It has been indicated that some receptors could still be activated without ligand. To test this, we determined the basal activity of all receptors in the absence of ligands by dual-reporter gene assay. As shown in Figure S3, we found that the intracellular basal cAMP levels of the four mutants had no significant differences compared with the wild type, suggesting that these mutations have no effect on the basal activity of receptors in cAMP production.

### Signaling Properties of pOX2R and Their Mutants With Ligands

**Figure 2:**
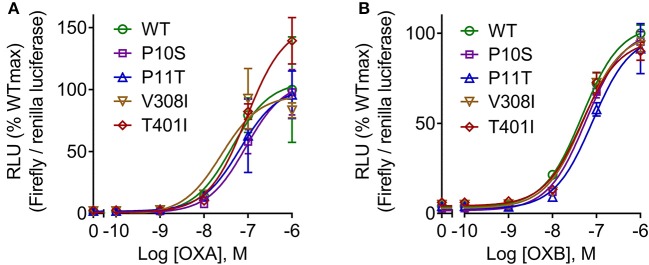
cAMP production of pOX2R wild type (WT) and four mutants under the stimulation of OXA **(A)** and OXB **(B)**. HEK293T cells were transiently transfected with the cloned pOX2R and mutants, and then cAMP production with two ligands was performed by dual-luciferase reporter gene assay. The maximum signaling of wild type was normalized as 100%, and the corresponding percentages are presented.

**Figure 3:**
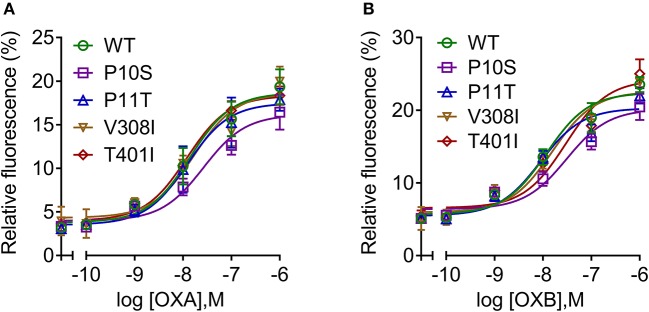
Intracellular calcium mobilization of pOX2R wild type (WT) and four mutants following stimulation with OXA **(A)** and OXB **(B)**. HEK293T cells were transiently transfected with the cloned pOX2R and mutants, and then calcium release with two ligands was measured by Fluo-4 AM assay.

To determine the response of cloned pOX2R and the four mutants to ligand stimulation in cAMP generation, the relative luciferase activities with two agonists from 10^−6^ to 10^−10^ mol/L OXA/OXB were evaluated. The results showed that pOX2R and the four mutants caused a dose-dependent increase of intracellular cAMP under the stimulation of two agonists, indicating that the expressed receptors are functional (Figure 2). Through analyzing the calculated EC_50_ (Table S2), we found that three of the mutants (P10S, P11T, and T401I) have an increased EC_50_ with OXA, suggesting that these mutants have a lower affinity to OXA compared with pOX2R and V308I (Figure 2A). However, under the stimulation of OXB, only P11T mutant showed a mildly decreased response (Figure 2B and Table S2). In addition, calcium release in HEK293T cells transfected with the cloned pOX2R and mutants following stimulation with OXA or OXB was determined. We found that only P10S showed decreased calcium release following stimulation with OXA or OXB, while the other three mutants exhibited no significant changes with the two ligands (Figures 3A,B and Table S2). These results demonstrated that the proline at position 10 and 11 plays an important role in the pOX2R signaling with OXA and OXB. Notably, although the ligand binding assays revealed that some mutants had relatively lower affinity compared with wild type, both receptors and mutants shared comparable maximal responses.

### pERK1/2, p38, and pCREB Levels of pOX2R and Their Mutants

**Figure 4:**
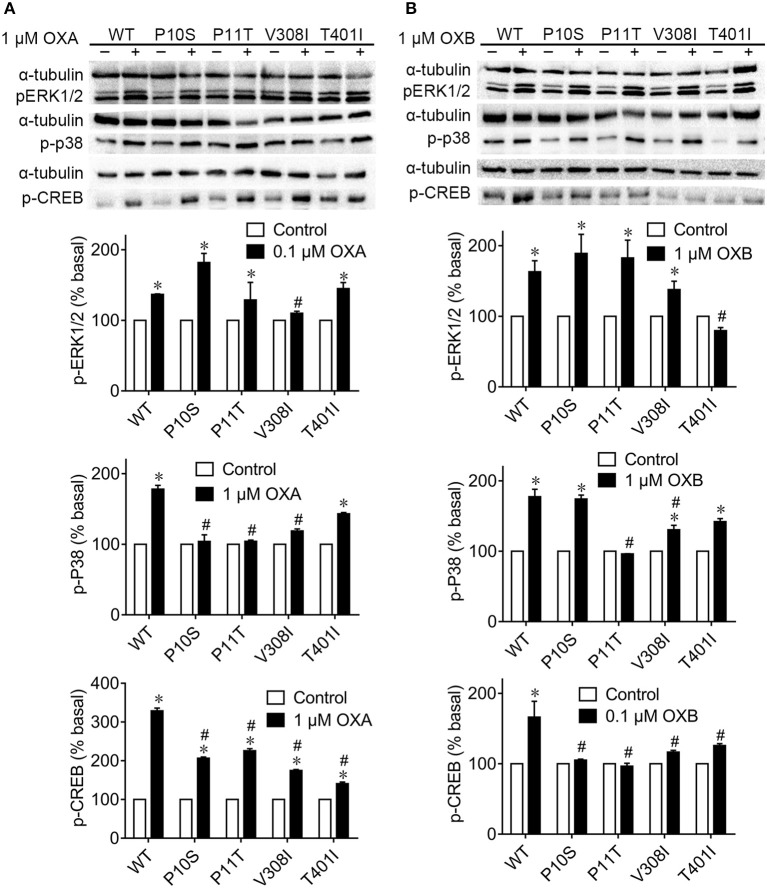
Phosphorylation levels of ERK1/2, p38, and CREB signaling of pOX2R wild type (WT) and mutants in the absence and presence of OXA **(A)** or OXB **(B)**. Transiently transfected HEK293T cells with receptors were stimulated with OXA or OXB, and the activation of three signals was determined by Western blot. *P < 0.05, significant difference compared to basal unstimulated receptor (normalized as 100%); ^#^*P* < 0.05, significant difference compared to the effect of orexin-stimulated wild type receptor.

To investigate the physiological activities of pOX2R wild type and mutants in the MAPK signaling pathway, Western blotting analysis to assess the phosphorylation levels of ERK1/2 and p38 was performed (Figure 4). We found that OXA/OXB caused significant phosphorylation of ERK1/2 for pOX2R wild type. Among the mutants, only V308I with OXA and T401I with OXB could not be activated and exhibited lower phosphorylation compared with the activated wild type.

Under the stimulation of OXA and OXB, the phosphorylation levels of p38 were significantly increased for pOX2R wild type. However, no remarkable phosphorylation levels of p38 protein were found in the P10S, P11T, and V308I mutants with agonist OXA. Compared with the activated pOX2R, two mutants, P11T and V308I, showed a significant decrease in the expression level of p-p38 under the action of the agonist OXB, and the mutant P11T was not significantly different from that when it was not stimulated. We then determined the phosphorylation levels of CREB under the stimulation of OXA/OXB (Figure 4). Consistent with previous reports for hOX2R (35, 36), the phosphorylation level of CREB in pOX2R significantly increased. Meanwhile, we found that the four mutants displayed a response to OXA stimulation with increased p-CREB. However, unlike OXA, OXB has no promotion effect on the four mutants in the phosphorylation of CREB.

## Discussion

In this study, we successfully cloned the pOX2R and obtained its full-length coding sequence. Phylogenetic analysis demonstrated that *pOX2R* showed the highest similarity with livestock sequences such as cattle and sheep and lowest similarity with zebrafish, followed by chicken. Homology models of OX2R from human and pig showed that only a few differences in protein 3D structures were observed, such as an additional α-helix in the non-transmembrane region for hOX2R. Consistent with several previous reports (37, 38), these findings suggested that mammalian OX2Rs have high conservation, particularly for the transmembrane region.

To elucidate the functions of some specific amino acids on the pharmacology of pOX2R, we constructed four mutants by PCR-mediated site-directed mutagenesis. Compared with the traditional methods, this method based on homologous recombination is more cost-effective and shows higher efficacy. Thus far, six hOX2R variants that are associated with early-onset narcolepsy in humans, P10S, P11T, C193S, I293V, V308I, and T401I, have been found (34, 39), but the pharmacological and physiological properties of these polymorphisms have not been determined in heterologous expression studies. In particular, the majority of GPCR polymorphisms are found in the loops and in the *N*- and *C*-terminal. Compared with the transmembrane core of GPCRs, the loops linking helices are much more variable and are considered not to be essential for the functions of the receptors (40). However, more evidence has demonstrated that amino acids located outside the binding cavity may also have consequences on the binding affinities between receptors and ligands. For example, P10S and P11T at the *N*-terminal may affect ligand and receptor binding or intracellular signal transduction through altering the receptor structure (41). Specifically, Thompson et al. found that response sensitivity of mutant P10S to OXA and OXB decreased 1.6- and 2.7-fold in COS-7 cells, respectively (41). Based on these results, we also constructed the P10S and P11T mutants of pOX2R in HEK293T. Of the six reported variants, only V308 at transmembrane region 6 is closer to the intracellular side and within the predicted transmembrane helix domain forming this cavity (42). Meanwhile, T401, located downstream of the palmitoylation site, may impact kinase or other protein binding. Combined with these facts and predictions, we speculated that these four amino acids may correlate with the binding activity of receptors or downstream signal transduction machinery. Therefore, these four pOX2R mutations, P10S, P11T, V308I, and T401I, were chosen for our subsequent investigations.

As a member of the GPCR superfamily, the conventional signaling pathway of OX2R involves stimulation of adenylyl cyclase activity, protein kinase A activation via cAMP coupling with Gs protein, and intracellular calcium mobilization coupled with Gq protein. Thus, we next determined the cAMP production and intracellular calcium concentrations.

Dual-luciferase reporter gene methods were performed to determine the cAMP production because they have higher sensitivity than the ELISA method (43). The results showed that three out of the four mutations displayed decreased cAMP production with OXA stimulation. However, only P11T had a lower cAMP production following stimulation with OXB, implying that OXB has a higher anti-interference ability than OXA. With respect to calcium release, consistent with previous results in hOX2R (41), P10S exhibited a decreased calcium release in response to both OXA and OXB. In addition to the classical Gs-cAMP and Gq-calcium signaling pathway, several other pathways, including the mitogen-activated protein kinase (MAPK) and proteinkinase (PKC) signaling pathways, may also be activated under OX2R stimulation. Among the MAPK pathways, the ERK1/2 pathway plays an important role in cell proliferation, differentiation, migration senescence, and apoptosis (44). However, p38 protein exerts analgesic effects on neuropathic pain and other chronic pain through triggering various intracellular responses (45). It has been proven that OXA/OXB could activate the phosphorylation of ERK1/2 and p38 in a dose- and time-dependent manner and that this process is independent of Gq/PLC/PKC and PKA pathways (46). Our subsequent Western blotting analysis demonstrated that OXA and OXB activated ERK1/2 and p38 proteins of pOX2R and increased their phosphorylated protein expression, which was consistent with the hOX2R. Considering the decreased cAMP generation and p-p38 for P11T, we thus concluded that this mutant may affect the conduction of the Gs/AC/cAMP/PKA pathway.

It has been indicated that the activation of CREB protein is highly dependent on PKC; thus, the level of p-CREB can reflect the activation of PKC to some extent (35). It should be noted that CREB could be activated by various intracellular signaling pathways, including the cAMP, Ca^2+^-CaMK, Ras/ERK, and P13/AKt signaling pathways; thus, its activation does not always reflect the intracellular cAMP levels (47, 48). In CHO cells, OXA and OXB have been proven to cause the phosphorylation of CREB for 40 min. Of note, higher concentrations of agonists were required for p-CREB compared to pERK1/2 (36). Our results showed that the cloned pOX2R also induced the phosphorylation of CREB under the stimulation of OXA/OXB. However, the expression of p-CREB in four mutations significantly decreased compared with wild type, indicating that these mutations seriously affected the activation of CREB with ligands. We reasoned that these four mutants may affect PKC upstream kinase activity. However, in the current state of knowledge, the underlying mechanisms are still unknown and need more investigation.

In conclusion, functional pOX2R and four mutants were successfully constructed and expressed in HEK293T cells. Subsequently, we investigated their signaling properties involving cAMP generation, calcium release, and the phosphorylation of ERK1/2, p38, and CREB and their signaling efficacies under the stimulation of two ligands. Results demonstrated that these mutants displayed decreased reactivity in varying degrees, suggesting the critical roles of these amino acids in the binding activity of receptors. Taken together, these findings provide new insight into the structure–activity relationship and physiological roles of pOX2R.

## Data Availability Statement

The datasets generated for this study are available from the corresponding authors Zhiqiang Wang (zqwang@yzu.edu.cn) or Yuan Liu (liuyuan2018@yzu.edu.cn) on reasonable request.

## Author Contributions

ZW and YL designed the experiments. ML and TM performed the experiments. ML, TM, and HZ analyzed the results. ML and YL wrote the manuscript. All authors reviewed the manuscript.

### Conflict of Interest

The authors declare that the research was conducted in the absence of any commercial or financial relationships that could be construed as a potential conflict of interest.
