# Supplementary material for: Pharmacological Characteristics of Porcine Orexin 2 Receptor and Mutants
Source: Front Endocrinol (Lausanne). 2020 Mar 31;11:132. doi: 10.3389/fendo.2020.00132 (PMC7136461; doi:10.3389/fendo.2020.00132)
Supplement: Supplementary file 1 [file Data_Sheet_1.docx]

**Supplementary material**

**Supplementary Figures**

**
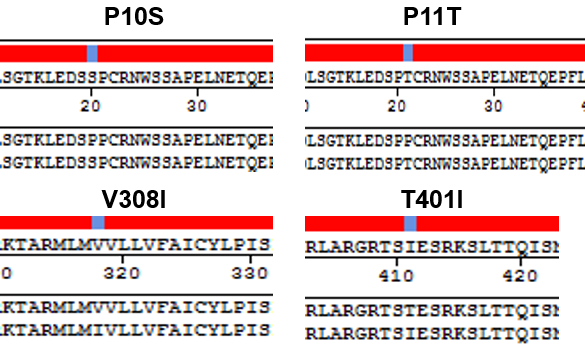
**

**Figure S1 Single amino acid mutation sites of pOX2R, including P10S, P11T, V308I and T401I.**

All obtained sequencing results are shifted down by 10 amino acids due to the addition of c-myc at *N* terminal.


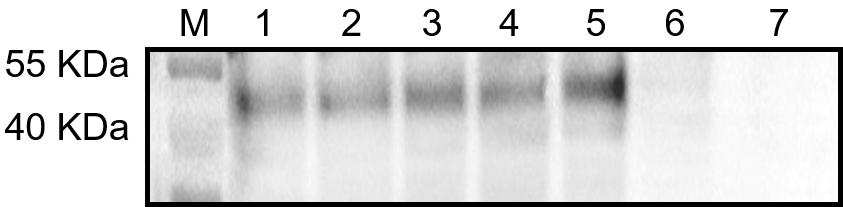


**Figure S2 Protein expression of pOX2R wild type and mutants in HEK293T by western blotting analysis.**

M, Protein Ladder; 1, transfected myc-pcDNA3.1(+)-pOX2R WT; 2, transfected myc-pcDNA3.1(+)-pOX2R P10S; 3, transfected myc-pcDNA3.1(+)-pOX2R P11T; 4, transfected myc-pcDNA3.1(+)-pOX2R V308I; 5, transfected myc-pcDNA3.1(+)-pOX2R T401I; 6, pcDNA3.1(+); 7, not transfected cells.

**

**

**Figure S3 Basal cAMP levels of the pOX2R wild type and four mutants by dual-luciferase reporter genes analysis.**

**Supplementary Tables**

**Table S1 Primers used in site-mutation of *pOX2R***

| Primer | Primer sequences (5’-3’) | Annealing temperature/℃ |
| --- | --- | --- |
| P10S | F: GCACCAAACTGGAGGACTCCTCCCCTTGTCG | 63 |
|  | R: AGGAGTCCTCCAGTTTGGTGCCGGATAGGTC |  |
| P11T | F: CCAAACTGGAGGACTCCCCCACTTGTCGCAA | 63 |
|  | R: TGGGGGAGTCCTCCAGTTTGGTGCCGGATAG |  |
| V308I | F: AAACAGCCCGGATGCTGATGATTGTGCTTTT | 60 |
|  | R: TCATCAGCATCCGGGCTGTTTTCCGTCTGGC |  |
| T401I | F: GGCCCGGGGACGGACGAGCATCGAAAGCCGA | 67 |
|  | R: ATGCTCGTCCGTCCCCGGGCCAGCCGATCCT |  |

**Table S2 Ligand potencies of pOX2R wild type and mutants in**

**cAMP production and calcium release.**

| Phenotype | cAMP production, EC_50_ (×10^-8^ M) | | Calcium release, EC_50_ (×10^-8^ M) | |
| --- | --- | --- | --- | --- |
|  | OXA | OXB | OXA | OXB |
| WT | 3.72 ± 0.88 | 4.62 ± 0.45 | 1.38 ± 0.16 | 1.17 ± 0.59 |
| P10S | 7.39 ± 2.07* | 5.17 ± 0.45 | 2.64 ± 0.23* | 3.07 ± 0.29* |
| P11T | 6.67 ± 1.27* | 8.18 ± 1.79* | 1.23 ± 0.10 | 2.85 ± 1.24 |
| V308I | 7.14 ± 2.04 | 3.93 ± 0.78 | 1.69 ± 0.28 | 1.54 ± 0.26 |
| T401I | 10.56 ± 2.44* | 4.51 ± 0.01 | 1.20 ± 0.09 | 2.96 ± 0.82 |

EC_50_: 50% effective concentration; **P* < 0.05, significant difference.
